# Maternal excessive weight gain as a potential risk factor for prolonged labor in Japanese pregnant women: The Japan Environment and Children’s Study

**DOI:** 10.1371/journal.pone.0306247

**Published:** 2024-07-03

**Authors:** Satoshi Shinohara, Sayaka Horiuchi, Reiji Kojima, Ryoji Shinohara, Sanae Otawa, Megumi Kushima, Kunio Miyake, Hideki Yui, Tadao Ooka, Yuka Akiyama, Hiroshi Yokomichi, Zentaro Yamagata, the Japan Environment and Children’s Study Group

**Affiliations:** 1 Department of Obstetrics and Gynecology, Yamanashi Prefectural Central Hospital, Kofu, Yamanashi, Japan; 2 Center for Birth Cohort Studies, University of Yamanashi, Chuo, Yamanashi, Japan; 3 Department of Health Sciences, School of Medicine, University of Yamanashi, Chuo, Yamanashi, Japan; Universidade Federal de Sao Paulo, BRAZIL

## Abstract

**Aim:**

This study aimed to determine whether excessive maternal weight gain during pregnancy was associated with a higher risk of prolonged labor.

**Methods:**

We analyzed the data regarding maternal weight gain during pregnancy for the participants of Japan Environment and Children’s Study (JECS), which is an ongoing nationwide prospective birth cohort study in Japan. After excluding participants with multiple pregnancies, with deliveries before 37 or beyond 42 weeks of gestation, or who had undergone cesarean section, 71,154 (nulliparous, n = 28,442) Japanese women were included. Prolonged labor was defined by a cutoff ranking at the 95^th^ percentile and consequently defined as labor duration exceeding 12.7 h in multiparous women and exceeding 23.2 h in nulliparous women. These classifications were made according to labor curves established by the Japanese Society of Obstetrics and Gynecology Perinatal Committee developed in June 2021. Considering that no studies have conducted an investigation based on this new guideline, we analyzed the association between excessive maternal weight gain during pregnancy and prolonged labor by parity.

**Results:**

The overall incidence of prolonged labor was 10.2% (2,907/28,442) in nulliparous women and 6.1% (2,597/42,712) in multiparous women. Multivariable analysis indicated that excessive maternal weight gain was significantly associated with prolonged labor in nulliparous (adjusted odds ratio, 1.21; 95% confidence interval, 1.10–1.32) and multiparous women (adjusted odds ratio, 1.15; 95% confidence interval, 1.05–1.27). Kaplan–Meier survival analysis showed that as labor progressed, the percentage of women who had not yet delivered was higher among those with excessive maternal weight gain than among those with normal maternal weight gain in both the nulliparous (median labor duration 12.9 h vs 12.2 h, p<0.001) and multiparous (median labor duration 6.2 h vs 5.8 h, p<0.001) groups.

**Conclusion:**

Excessive maternal weight gain was significantly associated with prolonged labor in Japanese women.

## Introduction

Prolonged labor is a labor pattern that deviates from that observed in most patients who experience spontaneous vaginal births [1]. It is defined as a protraction (i.e., slower than normal progress) or arrest disorder (i.e., complete cessation of progress) and affects about 8% of all women who give birth [1]. This condition, which is associated with an increased incidence of maternal and fetal morbidities, is one of the most common complications of childbirth. For the mother, prolonged labor increases the risk of instrumental delivery employing forceps or vacuum devices, third- or fourth-degree perineal lacerations, postpartum urinary retention, and postpartum hemorrhage [2–6]. Similarly, for neonates, prolonged labor increases the risk of admission to the neonatal intensive care unit, respiratory distress syndrome, sepsis, and neonatal seizures [4, 5, 7]. In clinical practice, it is difficult to predict the risk of prolonged labor. Therefore, it is essential to fully understand the risk factors associated with prolonged labor and to develop appropriate treatment strategies. Prolonged labor may be associated with several factors such as nulliparity, older maternal age, increased maternal weight gain, use of regional anesthesia, short stature, fetal occiput in a posterior or transverse position, and high birth weight [2, 8–12]. Several studies have assessed the role of excessive maternal weight gain as a risk factor for prolonged labor, [9, 10, 13] indicating that excessive maternal weight gain increases the risk of prolonged labor in European countries and the United States [9, 10]. However, considering that maternal physique differs by race and ethnicity, whether this relationship is relevant to the Japanese population remains to be examined [14]. In particular, pregnant women in Japan are physically shorter and thinner than those in European countries and the United States [14]. Recently, a nationwide prospective birth cohort study reported that approximately 10.9% of pregnant women in Japan have a pre-pregnancy body mass index (BMI) of 25.0 kg/m^2^ or higher [15]. Therefore, an improved understanding of the association between maternal weight gain and prolonged labor may provide clinically useful information for perinatal management in Japan. Moreover, in June 2021, the Japanese Society of Obstetrics and Gynecology Perinatal Committee issued new recommendations on appropriate weight gain during pregnancy [16]. According to this new recommendation, excessive weight gain was defined as weight gain of >15 kg (pre-pregnancy BMI: < 18.5 kg/m^2^); >13 kg (pre-pregnancy BMI: 18.5 to < 25.0 kg/m^2^); >10 kg (pre-pregnancy BMI: 25.0 to < 30.0 kg/m^2^); and >5 kg (pre-pregnancy BMI: ≥ 30.0 kg/m^2^) [16]. However, there are no published studies on the relationship between the “new” criteria for appropriate weight gain during pregnancy and labor progression to date, and evidence supporting the guidelines for prolonged labor according to maternal weight gain remains insufficient in Japan. To further reduce the incidence of perinatal complications caused by labor, the association between maternal weight gain and labor duration in the Japanese population should be extensively investigated. Examining the association between maternal weight gain and labor duration in different countries and ethnic groups may provide new opportunities to elucidate pathways leading to better management of perinatal complications caused during labor. Moreover, studies on maternal weight gain and duration of labor among the Japanese population are important for the medical/obstetric community outside Japan.

Therefore, this study aimed to determine whether excessive maternal weight gain during pregnancy was associated with a higher risk of prolonged labor.

## Methods

### Study design, setting and participants

The JECS is an ongoing nationwide prospective birth cohort study. The study was conducted at 15 Regional Centers (Hokkaido, Miyagi, Fukushima, Chiba, Kanagawa, Koshin, Toyama, Aichi, Kyoto, Osaka, Hyogo, Tottori, Kochi, Fukuoka, and South Kyusyu/Okinawa) in Japan. Details of the JECS project have been described in previous studies [17–19]. This study was conducted in compliance with the Strengthening the Reporting of Observational Studies in Epidemiology statement for observational studies and recruited pregnant women between January 2011 and March 2014. The eligibility criteria for participation included women residing in the study areas at the time of recruitment, with expected delivery date after August 2011, who comprehended Japanese language, and who had completed a self-administered questionnaire.

A total of 104,062 fetal records were included in this cohort. We included only those mothers whose obstetric and demographic data were complete. Importantly, information that could identify individual participants during or after data collection was inaccessible. Exclusion criteria were as follows: 1) delivery before 37 weeks of gestation 2) delivery beyond 42 weeks of gestation 3) delivery by cesarean section 4) multiple pregnancies and 5) missing data.

### Ethics statement

The JECS protocol was reviewed and approved by the Ministry of the Environment’s Institutional Review Board on Epidemiological Studies and the Ethics Committees of all participating institutions (Ethical Number: No.100910001). Written informed consent was obtained from all participants. The JECS was conducted in accordance with the Declaration of Helsinki and other national regulations.

### Variables

#### Data collection

The study participants completed questionnaires at different time points throughout pregnancy that is, during the first, second, and third trimesters, and postpartum periods. Medical records at the time of registration and immediately after vaginal or cesarean section were transcribed by physicians, midwives, nurses, and research coordinators. Information on maternal demographic factors was obtained from questionnaires completed during pregnancy. Obstetric records included maternal age at delivery, infant sex, presence of gestational diabetes mellitus (GDM), presence of hypertensive disorder of pregnancy (HDP), use of assisted reproductive technology (ART), parity, gestational age at delivery, maternal stature, pre-pregnancy weight status, and body weight at the last prenatal visit within one week.

The obstetrician in charge at the time of labor induction or augmentation determined the dose and type of uterine contraction agents (oxytocin or prostaglandins) based on the Japanese obstetric practice guidelines [20]. The diagnosis of GDM was based on the following: at least one abnormal plasma glucose value (≥92, 180, and 153 mg/dL for fasting, 1-hour, and 2-hour plasma glucose concentration, respectively) after a 75 g OGTT [20]. HDP was defined as blood pressure ≥140/90 mmHg [21]. Pre-pregnancy BMI was calculated according to the World Health Organization standard (body weight [kg]/height [m^2^]). Furthermore, pre-labor BMI was defined as BMI within 1 week retrospective from the date of delivery.

ART was defined as in vitro fertilization or intracytoplasmic sperm injection. Instrumental delivery was defined as the use of forceps or vacuum extraction.

#### Exposure definitions

Pregnancy weight gain was calculated by subtracting the pre-pregnancy body weight from the body weight at the last prenatal visit. Prenatal check-ups in Japan are conducted once weekly from 36 to 39 weeks and twice weekly after 40 weeks [20]. Consequently, in term pregnancies, the body weight at the last prenatal visit was the weight measured within 7 days before delivery. Excessive maternal weight gain was defined according to the pre-pregnancy BMI.

#### Covariates

The following were considered as potential confounding factors: maternal obesity, [22] short maternal stature, [2] large-for-gestational-age (LGA) infants, [11, 12] epidural anesthesia during labor, [2] and advanced maternal age (≥35 years) [8] because these factors have been previously identified as risk factors for prolonged labor. In the epidemiology field, when selecting covariates, referring to previous knowledge is a widely used strategy [23, 24].

#### Outcomes

The period between admission for the onset of labor and full cervical dilation was defined as the time required for the first stage of labor [25–27]. The period between full cervical dilation and delivery of the baby was defined as the time required for the second stage of labor [25–27] The duration of labor was calculated as the sum of the first and second stages of labor [25–27] The Japanese Society of Obstetrics and Gynecology Perinatal Committee developed a new labor curve in June 2021 [25]. According to this study, prolonged labor was defined by a cut-off value at the 95^th^ percentile ranking and was consequently defined as a duration of labor exceeding 12.7 h in multiparous women and a duration of labor exceeding 23.2 h in nulliparous women [25]. Therefore, we used this labor curve as a reference in this study.

### Data sources and measurement

This study used the jecs-ta-201901930-qsn dataset, which was released in October 2019 and revised in February 2020. Information regarding maternal weight gain was obtained from the jecs-ta-201901930-qsn dataset. When creating the jecs-ta-201901930-qsn dataset, medical records were transcribed by doctors, research coordinators, nurses, and midwives.

### Study size

**Fig 1:**
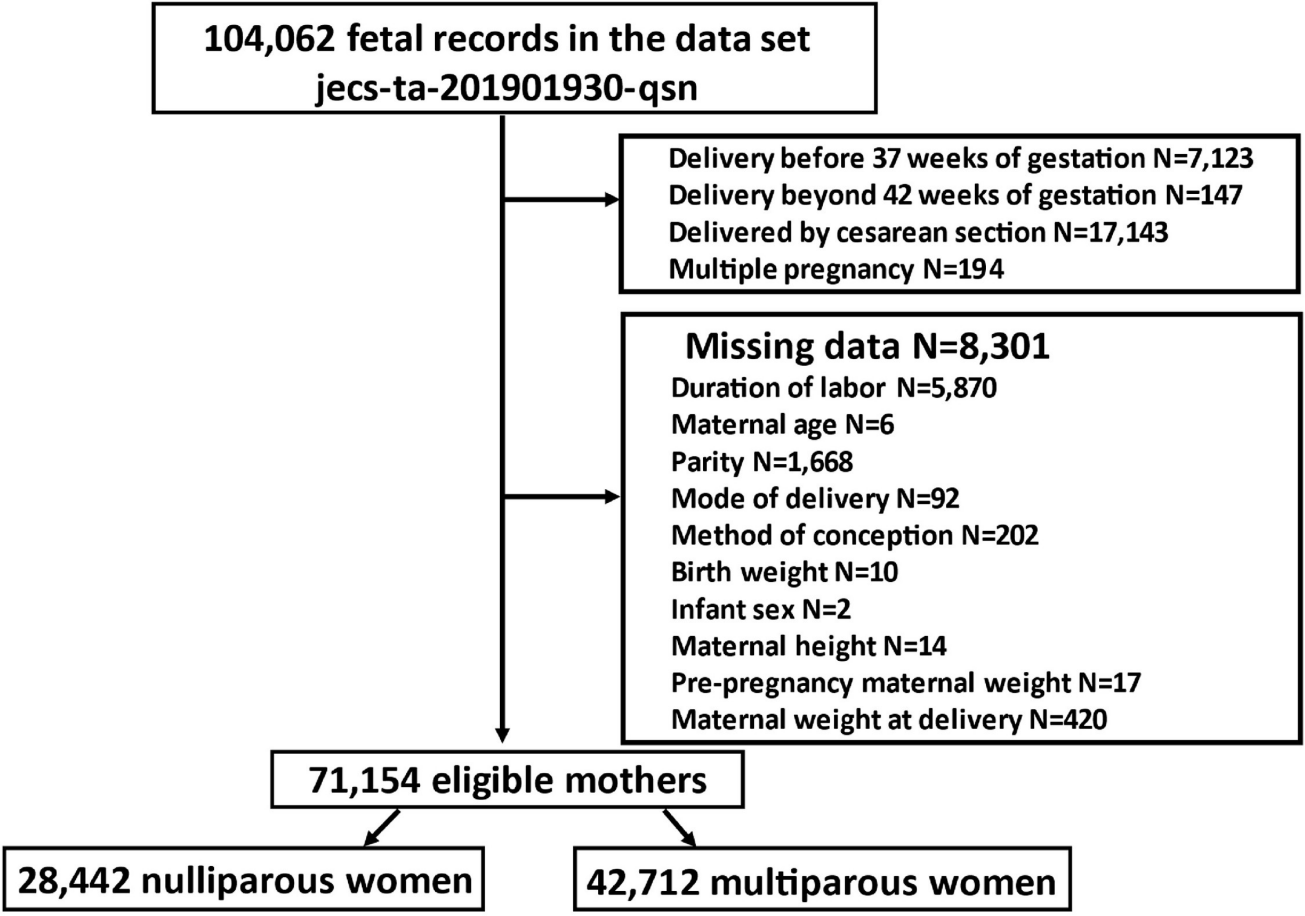
Flow chart of the selection of the pregnant women.

A total of 104,062 fetal records were included in this cohort study. We excluded women who delivered before 37 weeks of gestation (n = 7,123), delivered beyond 42 weeks of gestation (n = 147), delivered by cesarean section (n = 17,143), and had multiple pregnancies (n = 194). Of the 17,143 women who underwent a cesarean section, 1,717 were diagnosed with arrest of labor. We also excluded women with missing data regarding various parameters including duration of labor, n = 5,870; maternal age, n = 6; parity, n = 1,668; mode of delivery, n = 92; method of conception, n = 202; birthweight, n = 10; infant sex, n = 2; maternal height, n = 14, pre-pregnancy maternal weight, n = 17; and maternal weight at delivery, n = 420 (Fig 1).

### Quantitative variables

Women were categorized into four groups (G) for analysis, according to their pre-pregnancy BMI: G1 included women with BMI <18.5 kg/m^2^; G2 included women with BMI 18.5 to <25.0 kg/m^2^; G3, included women with BMI 25.0 to <30.0 kg/m^2^; and G4 included women with BMI ≥30.0 kg/m^2^. Excessive weight gain was defined as weight gain of >15 kg for G1, >13 kg for G2, >10 kg for G3, and >5 kg for G4 [16]. Women were classified as obese (BMI ≥25.0 kg/m^2^) or non-obese (BMI <25.0 kg/m^2^), according to the Japan Society of Obstetrics and Gynecology Guidelines for Obstetrical Practice [20] According to a previous study, short stature was defined as a height of <150 cm [28] We defined LGA as a birth weight above the 10^th^ percentile at each gestational week [29].

### Statistical analysis

Student’s t-test and the *χ*^*2*^ test (or Fisher’s exact test when the expected frequency was <5) were used to evaluate the effect of potential confounding factors on prolonged labor. Multivariate logistic regression analysis was performed by adding all covariates and evaluating association between excessive maternal weight gain and prolonged labor. Results were reported as odds ratios (ORs) and 95% confidence intervals (CIs). Finally, we performed the Kaplan–Meier analysis and log-rank test to examine statistical differences based on the influence of excessive maternal weight gain on the duration of labor. All analyses were performed using IBM SPSS Statistics for Windows, version 25 (IBM Corp., Armonk, NY, US). The significance level was set at p < 0.05

## Results

A total of 71,154 women were included in the study. The mean maternal age and pre-pregnancy BMI were 30.9 ± 5.0 years and 21.1 ± 3.1 kg/m^2^, respectively, with 28,442 nulliparous women (40.0%), 36,269 male infants (51.0%), 1,477 women with GDM (2.1%), 1,386 women with HDP (1.9%), and 6,075 LGA infants (8.5%). Clinical characteristics of the women included in this study are shown in Table 1. The overall incidence rate of prolonged labor was 7.7% (5,504/71,154), and the mean duration of labor was 8.45 ± 7.19 h. Of the 15,996 women who showed excessive weight gain during pregnancy, 13,262 (82.9%) had a pre-labor BMI of ≥25.0 kg/m^2^.

**Table 1:** Baseline obstetric and demographic data of the study population.

	Excessive weight gain n = 15,996	Normal weight gain n = 55,158	p-value
**Gestational age, weeks**	39.4±1.0	39.1±1.1	<0.001
**Nulliparous**	7,078 (44.2)	21,364 (38.7)	<0.001
**Maternal height, cm**	158.9±5.2	158.2±5.3	<0.001
**Pre-pregnancy BMI, kg/m** ^**2**^	21.8±3.5	20.8±2.9	<0.001
**Pre-labor BMI, kg/m** ^**2**^	27.8±3.2	24.5±2.7	<0.001
**ART**	227 (1.4)	1,342 (2.4)	<0.001
**Birth weight, g**	3,219±363.9	3,044±349.2	<0.001
**GDM**	250 (1.6)	1,227 (2.2)	<0.001
**HDP**	524 (3.3)	862 (1.6)	<0.001
**Instrumental delivery**	1,236 (7.7)	3,881 (7.0)	0.003
**Pre-treatment with oxytocin**	3,959 (24.7)	10,879 (19.7)	<0.001
**Pre-treatment with PGF2α**	418 (2.6)	1,012 (1.8)	<0.001
**Use of epidural anesthesia**	346 (2.2)	1,163 (2.1)	0.67
**Male infant**	8,344 (52.2)	27,925 (50.6)	0.001
**LGA infant**	2,455 (15.3)	3,620 (6.6)	<0.001
**Duration of labor, hour**	9.2±7.6	8.2±7.1	<0.001
**Prolonged labor**	1,455 (9.1)	4,049 (7.3)	<0.001

Values are shown as average ± standard deviation or numbers (%).

BMI, body mass index; ART, assisted reproductive technology; HDP, hypertensive disorder of pregnancy; GDM, gestational diabetes mellitus; PGF, prostaglandin F; LGA, large for gestational age

Among continuous variables, gestational age, maternal height, pre-pregnancy BMI, pre-labor BMI, birth weight, and duration of labor were significantly higher in the maternal excessive weight gain group than in the maternal non-excessive weight gain group. Moreover, among categorical variables, nulliparity, HDP, instrumental delivery, pre-treatment with oxytocin, pre-treatment with PGF2α, male infant, LGA infant, and prolonged labor were significantly higher whereas ART and GDM were significantly lower in the maternal excessive weight gain group than in the maternal non-excessive weight gain group (Table 1). We described the categorization of the women into the different groups that define excessive weight gain to better understand the study results (Table 2).

**Table 2:** Classification of women into the different groups that define excessive weight gain.

Nulliparity	G1	G2	G3	G4
**Excessive weight gain n = 7,078**	653 (9.2)	5,506 (77.8)	677 (9.6)	242 (3.4)
**Normal weight gain n = 21,364**	4,587 (21.5)	15,551 (72.8)	1,024 (4.8)	202(0.9)
**Multiparity**	G1	G2	G3	G4
**Excessive weight gain n = 8,918**	677 (7.6)	6,552 (73.5)	1,183 (13.3)	506 (5.6)
**Normal weight gain n = 33,794**	5,995 (17.7)	24,984 (73.9)	2,339 (6.9)	476 (1.5)

Values are shown as numbers (%).

Pre-pregnancy BMI: G1, < 18.5 kg/m^2^; G2, 18.5 to < 25.0 kg/m^2^; G3, 25.0 to < 30.0 kg/m^2^; and G4 (≥ 30.0 kg/m^2^).

Excessive weight gain was defined as >15 kg for G1; >13 kg for G2; >10 kg for G3; and >5 kg for G4

Of the 6,649 obese pregnant women in G3 or G4, 2,608 (39.3%) showed excess weight gain, and, of the 2,145 nulliparous obese pregnant women in G3 or G4, 919 (42.8%) showed excess weight gain. Furthermore, of the 4,504 multiparous obese pregnant women in G3 or G4, 1689 (37.5%) showed excess weight gain.

According to the jecs-ta-201901930-qsn dataset used in our analysis, which was created from the JECS, of the 17,143 women who delivered by cesarean section (i.e., women who were excluded from our analysis), 1,717 underwent cesarean section due to labor arrest. Additionally, among 1,695 women who delivered via cesarean section, excluding 22 women who delivered via cesarean section with missing data on maternal weight gain, 703 (41.5%) had excessive weight gain during pregnancy. In contrast, among the 71,154 women in our analysis, 15,996 (22.5%) had excessive weight gain during pregnancy. In other words, in the JECS population, women who gained excess weight during pregnancy were at a significantly higher risk of undergoing a cesarean section than women who gained normal weight during pregnancy (p < 0.001).

### Relationship between maternal excessive weight gain and prolonged labor in nulliparity

Clinical characteristics of the 28,442 nulliparous women included in this study are shown in Table 3. The overall incidence rate of prolonged labor was 10.2% (2,907/28,442), and the mean duration of labor was 12.35 ± 8.86 h. The correlation coefficient between maternal weight gain and the duration of labor was estimated as 0.05 (G1), 0.05 (G2), 0.03 (G3) and 0.00 (G4). Of the 7,078 women who showed excessive weight gain during pregnancy, 5,812 (82.1%) had a pre-labor BMI of ≥25.0 kg/m^2^.

**Table 3:** Baseline obstetric and demographic data of nulliparous women in the study population.

	Excessive weight gain n = 7,078	Normal weight gain n = 21,364	p-value
**Gestational age, weeks**	39.5±1.0	39.3±1.1	0.08
**Maternal height, cm**	158.9±5.3	158.2±5.3	0.48
**Pre-pregnancy BMI, kg/m** ^**2**^	21.5±3.3	20.6±2.8	<0.001
**Pre-labor BMI, kg/m** ^**2**^	27.6±3.1	24.3±2.6	<0.001
**ART**	147 (2.1)	871 (4.1)	<0.001
**Birth weight, g**	3162.7±351.5	2990±338.3	<0.001
**GDM**	117 (1.7)	473 (2.2)	0.004
**HDP**	294 (4.2)	21,364 (2.1)	<0.001
**Instrumental delivery**	907 (12.8)	2,635 (12.3)	0.29
**Pre-treatment with oxytocin**	2,300 (32.5)	5,950 (27.9)	<0.001
**Pre-treatment with PGF2α**	239 (3.4)	524 (2.5)	<0.001
**Use of epidural anesthesia**	191 (2.7)	532 (2.5)	0.33
**Male infant**	3,655 (50.2)	10,757 (50.4)	0.06
**LGA infant**	1,240 (17.5)	1,647 (7.7)	<0.001
**Duration of labor, hours**	12.9±9.0	12.2±8.8	0.001
**Prolonged labor**	832 (11.8)	2,075 (9.7)	<0.001

Values are shown as average ± standard deviation or as numbers (%).

BMI, body mass index; ART, assisted reproductive technology; HDP, hypertensive disorder of pregnancy; GDM, gestational diabetes mellitus; PGF, Prostaglandin F; LGA, large for gestational age.

**Fig 2:**
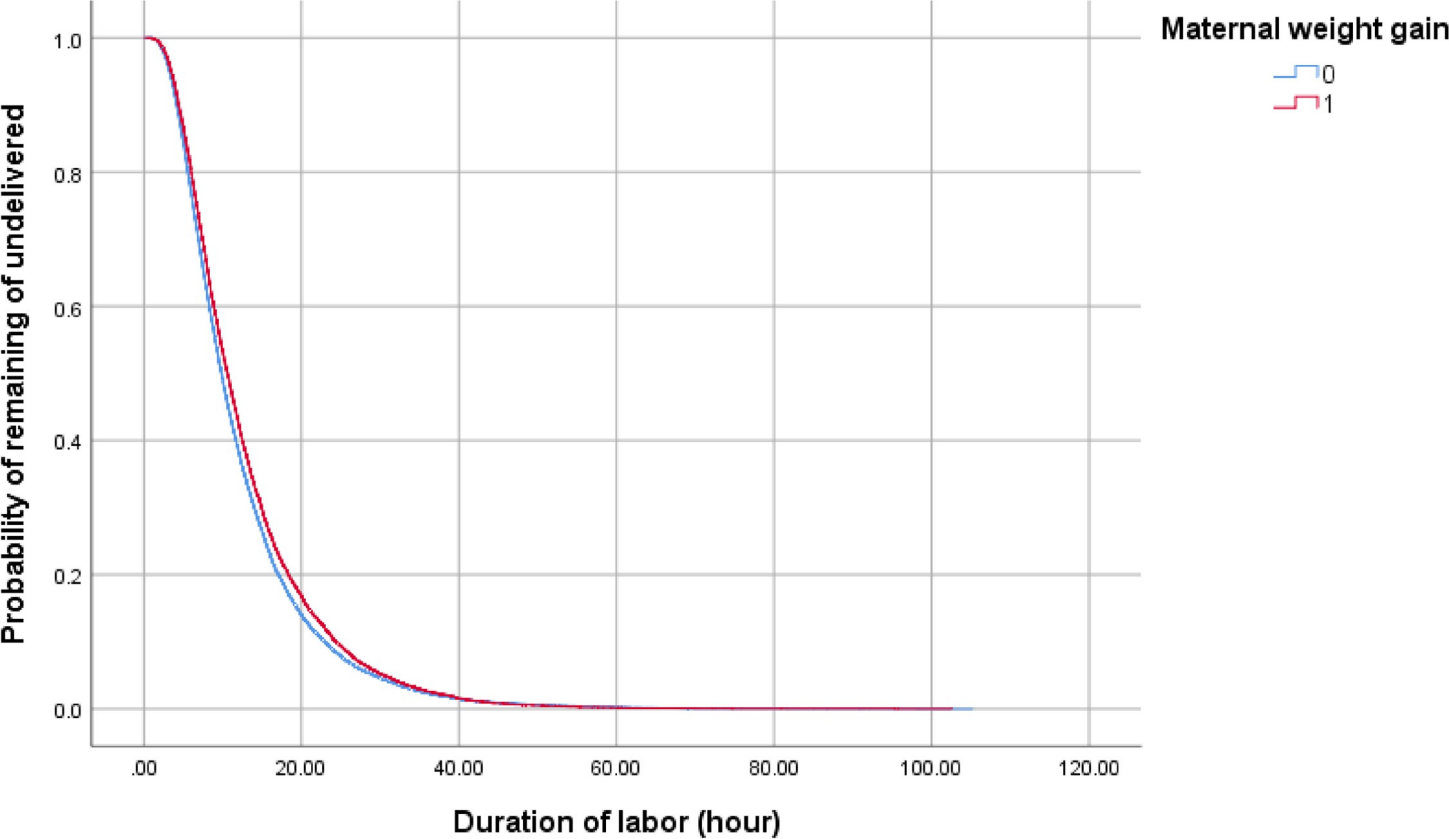
Kaplan–Meier curves. Kaplan–Meier curves of the duration of labor according to maternal weight gain in nulliparous women. 1: Excessive weight gain group 0: Normal weight gain group.

In multivariable analyses, excessive maternal weight gain (aOR, 1.21; 95% CI, 1.10–1.32; p<0.001) was associated with prolonged labor (Table 4). Using the Kaplan–Meier survival analysis (Fig 2), as labor progressed, the percentage of nulliparous women whose duration of labor was prolonged was larger for women with excessive maternal weight gain when they delivered their neonates compared to that of women with normal maternal weight gain (median duration of labor 12.9 h vs 12.2 h, p <0.001).

**Table 4:** Association between maternal excessive weight gain and prolonged labor in nulliparous women.

	Maternal weight gain during pregnancy	Crude	p-value	Adjusted	p-value
		OR [95%CI]		OR [95%CI]	
**Prolonged labor**	Excessive weight gain	1.24 [1.13–1.35]	<0.001	1.21 [1.10–1.32]	**<0.001**
	Normal weight gain	Reference		Reference	

*Regression analysis was performed after adjusting for maternal obesity, short maternal stature, large-for-gestational-age infants, epidural anesthesia during labor, and advanced maternal age. The variance inflation factor ranged between 1.003 and 1.038.

OR, odds ratio; CI, confidence interval

### Relationship between maternal excessive weight gain and prolonged labor in multiparity

Table 5 summarizes the obstetric and demographic data of the 42,712 multiparous women who were enrolled in the study. The overall incidence rate of prolonged labor was 6.1% (2,597/42,712). The mean duration of labor was 5.85 ± 4.13 h. The correlation coefficient between maternal weight gain and the duration of labor was estimated as 0.06 (G1), 0.05 (G2), 0.03 (G3) and 0.06 (G4). Of the 8,918 women who showed excessive weight gain during pregnancy, 7,450 (83.5%) had a pre-labor BMI of ≥25.0 kg/m^2^.

**Table 5:** Baseline obstetric and demographic data of multiparous women in the study population.

	Excessive weight gain n = 8,918	Normal weight gain n = 33,794	p-value
**Gestational age, weeks**	39.3±1.0	39.1±1.0	<0.001
**Maternal height, cm**	158.9±5.2	158.1±5.2	0.09
**Pre-pregnancy BMI, kg/m** ^**2**^	22.1±3.7	21.0±3.0	<0.001
**Pre-labor BMI, kg/m** ^**2**^	28.0±3.3	24.6±2.7	<0.001
**ART**	80 (0.90)	471 (1.4)	<0.001
**Birth weight, g**	3263.3±367.3	3078.4±351.7	<0.001
**GDM**	133 (1.5)	754 (2.2)	<0.001
**HDP**	230 (2.6)	413 (1.2)	<0.001
**Instrumental delivery**	329 (3.7)	1,246 (3.7)	0.99
**Pre-treatment with oxytocin**	1,659 (18.6)	4,929 (14.6)	<0.001
**Pre-treatment with PGF2α**	179 (2.0)	488 (1.4)	<0.001
**Use of epidural anesthesia**	155 (1.7)	631 (1.9)	0.42
**Male infant**	4,689 (52.6)	17,168 (50.8)	0.003
**LGA infant**	1,215 (13.6)	1,973 (5.8)	<0.001
**Duration of labor, hour**	6.2±4.4	5.8±4.1	<0.001
**Prolonged labor**	623 (7.0)	1,974 (5.8)	<0.001

Values are shown as average ± standard deviation or as numbers (%).

BMI, body mass index; ART, assisted reproductive technology; HDP, hypertensive disorder of pregnancy; GDM, gestational diabetes mellitus; PGF, Prostaglandin F; LGA, large for gestational age.

**Fig 3:**
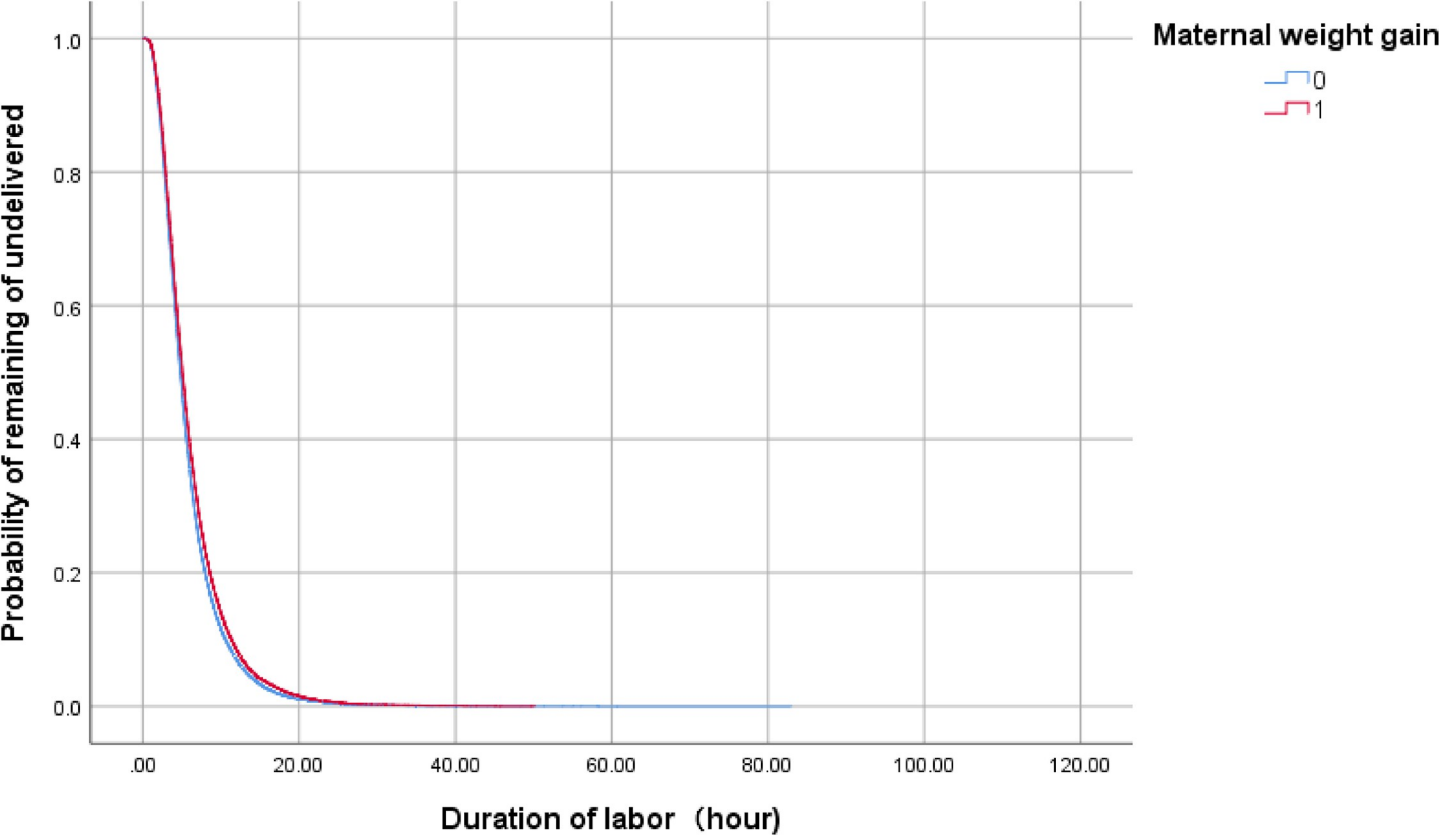
Kaplan–Meier curves. Kaplan–Meier curves of the duration of labor according to maternal weight gain in multiparous women. 1: Excessive weight gain group 0: Normal weight gain group.

In multivariable analyses, excessive maternal weight gain (aOR, 1.15; 95% CI, 1.05–1.27; p = 0.004) was associated with prolonged labor (Table 6). Using the Kaplan–Meier survival analysis (Fig 3), as labor progressed, the percentage of women whose duration of labor was prolonged was larger for multiparous women with excessive maternal weight gain when they delivered their neonates compared to that of women with normal maternal weight gain (median duration of labor 6.2 h vs 5.8 h, p < 0.001).

**Table 6:** Association between maternal excessive weight gain and prolonged labor in multiparous women.

	Maternal weight gain during pregnancy	Crude	p-value	Adjusted	p-value
		OR [95%CI]		OR [95%CI]	
**Prolonged labor**	Excessive weight gain	1.21 [1.10–1.13]	<0.001	1.15 [1.05–1.27]	0.004
	Normal weight gain	Reference		Reference	

*Regression analysis was performed after adjusting for maternal obesity, maternal short stature, large for gestational age infants, epidural anesthesia during labor, and advanced maternal age. The variance inflation factor ranged between 1.001 and 1.037.

OR, odds ratio; CI, confidence interval

## Discussion

This study’s findings suggests that excessive maternal weight gain significantly affected the duration of labor in nulliparous and multiparous women. Excessive maternal weight gain was significantly associated with prolonged labor after adjusting for potential confounding factors, and was inversely correlated with the duration of labor in all pregnant women.

Several factors are assumed to contribute in the pathophysiology of prolonged labor induced by excessive maternal weight gain. First, the more weight gained during pregnancy, the more the secretion of adipokine leptin, which is primarily released by white adipose tissue [22]. Leptin stimulates the release of prostaglandin E_2_ from the adipose and placental tissues through inflammatory signaling pathways [22]. Chronic elevation of prostaglandin E_2_ levels associated with meta-inflammation in late pregnancy in women who gain excess weight during pregnancy may reduce the sensitivity of maternal tissues to prostaglandin E_2_ during parturition activation [22]. Second, leptin inhibits collagen degradation and cervical cell apoptosis in vitro. These two actions may inhibit cervical maturation in obese women [22]. Furthermore, the fact that leptin stimulates cervical collagen synthesis in late pregnancy may explain the clinically observed decrease in cervical maturation at delivery in obese women [22]. Finally, it has also been speculated that oxytocin receptors may be influenced by maternal obesity. According to a previous study, oxytocin receptor mRNA was decreased in myometrial biopsies of term pregnancies in obese women at delivery [30].

To date, only a few studies have focused on the association between excessive maternal weight gain and the duration of labor [9, 10, 13]. In the United States, a retrospective chart review of 104 women revealed that weight gained during pregnancy influences progress and intervention during the second stage of labor [10]. The previous study differed from our analysis in that it considered only the association between maternal weight gain and the second stage of labor without examining the association between maternal excessive weight gain and the duration of labor in terms of the potential confounding factors for prolonged labor [10]. Another retrospective case-control study conducted in Finland reported that weight gain during pregnancy did not show an association with the duration of the first stage of labor; in contrast, the second stage of labor was significantly shorter in women with weight gain <5 kg during pregnancy [9]. Their retrospective case-control study, which divided 191 eligible women into three groups based on weight gain of >20 kg, 5–20 kg, and <5 kg during pregnancy, differed from ours [9]. The differences pertained to how the eligible women were classified and the objective variables used. However, compared to these previous studies, [9, 10, 13] our study analyzed many risk factors for prolonged labor and included a much larger sample size.

In Japan, a multicenter retrospective cohort study reported that excessive gestational weight increases the risk of labor arrest [31]. This study’s result is consistent with our finding that excessive maternal weight gain was significantly greater in the group of women who delivered by cesarean section due to labor arrest than in the group of women who delivered vaginally.

The duration of labor was found to differ by 0.7 h (42 minutes) in nulliparity and by 0.4 h (24 minutes) in multiparity in the present results. Although the log-rank test shows a significant difference between maternal weight gain and the duration of labor in nulliparous or multiparous women, the lines for the normal weight and the excessive weight gain groups are almost merging (Figs 2 and 3). Moreover, the correlation coefficient with each category of pre-pregnancy BMI between maternal weight gain and the duration of labor ranged from 0.00 to 0.06.

Classification by pre-pregnancy BMI shows that maternal weight gain and the duration of labor were not strongly correlated. Therefore, in the clinical setting, whether this result supports the hypothesis that the percentage of women whose labor duration was larger for women with excessive maternal weight gain when they delivered their neonates than in those with normal maternal weight gain in nulliparous or multiparous women requires careful consideration.

On the other hand, the 1,695 women who delivered via cesarean section also included those women who would have delivered vaginally had they taken more time. Excluding these women may have led to underestimating the relationship between maternal weight gain and prolonged labor, especially when conducting survival analysis. To alleviate some underestimation when depicting survival curves, we defined an event as vaginal delivery, whereas caesarean delivery due to labor arrest was censored. However, according to the jecs-ta-201901930-qsn dataset used in our analyses, for pregnant women undergoing cesarean section, there is no information indicating time between labor onset and the decision to perform a cesarean section due to labor arrest. Therefore, when interpreting the results of this study, it is important to note that the effect of excessive maternal weight may be estimated by excluding women who delivered babies by cesarean section not only for labor arrest but also for other reasons such as previous cesarean section, HDP, previa placenta, non-reassuring fetal status, intrauterine infection, cephalopelvic disproportion. In the excessive maternal weight group, we performed Kaplan–Meier analysis and a log-rank test to examine statistical differences based on the influence of obesity and maternal age on labor duration. With regard to advanced maternal age, previous studies have shown that with age, the myometrium, in particular, is less effective and/or responsive to oxytocic agents or prostaglandins [32]. Moreover, several reports indicated that oxytocin use was higher in older women than in the younger women during the course of parturition [32–34]. The percentage of nulliparous women with a prolonged labor duration was not larger for women with obesity compared to those without obesity (median duration of labor 12.4 h vs 13.0 h, p = 0.07). In contrast, there was an increased percentage of multiparous women with prolonged labor duration for women with obesity than in those without obesity (median duration of labor 6.4 h vs 6.1 h, p = 0.044). Further, the percentage of nulliparous and multiparous women with prolonged labor duration was not significant for women with advanced age when compared to that in younger women (median duration of labor 13.0 h vs 12.9 h, p = 0.79 and median duration of labor 6.1 h vs 6.2 h, p = 0.21). In a subgroup analysis of obesity and older age in the excessive maternal weight group, we assumed that they may show prolonged labor. However, three of the four analyses did not find significant differences, which suggests that it is necessary to carefully assess whether this association in multiparous women is also clinically significant. The exclusion of cesarean section cases may have led to an underestimation of the study results.

The strength of the study includes its large sample size, as nationwide cohort study data was included. The JECS, Japan’s largest nationwide birth cohort study, included 100,000 participants and is considered representative of the general population. Furthermore, the outcome measurements were reliable based on pregnancy and delivery information from medical records transcribed by physicians, midwives/nurses, and/or research coordinators.

However, our study had some limitations. First, we did not evaluate all potential risk factors for prolonged labor, such as uterine abnormality [35] and fetal occiput in the posterior or transverse position [11, 12]. Certain unmeasured confounders may be associated with prolonged labor. Second, we could only extract from the jecs-ta-201901930-qsn dataset, the total time for the first and second stages of labor as the duration of labor. According to previous reports, [9, 10, 13, 30] there is no general consensus on whether excessive maternal weight gain is associated with labor’s first or second stages. Depending on whether the first or second stage of labor is more prolonged, medical interventions could differ [36–40]. Investigating whether excessive maternal weight gain is more strongly associated with the first or second stage of labor could provide more useful information for perinatal management. Finally, although this is not a limitation, ART and GDM were significantly lower in the maternal excessive weight gain group than in the maternal non-excessive weight gain group in our analysis. There is no general opinion to date, but several reports examine the association between ART or GDM and excessive maternal weight gain [41–42]. The association between ART or GDM and maternal excessive weight gain could be an important perinatal topic. Therefore, we would like to examine these associations in detail in the future.

In conclusion, excessive maternal weight gain was significantly associated with prolonged labor in nulliparous and multiparous Japanese women. It is likely that the impact of maternal weight gain on the duration of labor might have been underestimated due to the exclusion of cesarean section cases, especially those caused by labor arrest. Although the results of this study indicate the need to provide pregnant women with counseling sessions about the possible effects of excessive maternal weight gain during pregnancy to prevent prolonged labor as well as other adverse perinatal events, they are inconclusive in that excessive weight gain during pregnancy causes prolonged labor. Instead, it may be better to counsel patients about the importance of other risk factors that cause prolonged labor. However, the finding that excessive maternal weight gain has a significant impact on prolonged labor, even if underestimated, is very important. We hope that the results of this study will be sufficient grounds for further research aimed at reducing perinatal complications.
